# Gender-based violence and engagement in biomedical HIV prevention, care and treatment: a scoping review

**DOI:** 10.1186/s12889-019-7192-4

**Published:** 2019-07-08

**Authors:** Anna M. Leddy, Ellen Weiss, Eileen Yam, Julie Pulerwitz

**Affiliations:** 1 Division of Prevention Science, Center for AIDS Prevention Studies, University of California, San Francisco, 550 16th St., 3rd Floor, San Francisco, CA 94158 USA; 2 Population Council, 4301 Connecticut Ave. NW, # 280, Washington, DC 20008 USA

**Keywords:** Gender-based violence, Intimate partner violence, Pre-exposure prophylaxis, HIV care and treatment

## Abstract

**Background:**

While gender-based violence (GBV) has been shown to increase women’s risk of HIV acquisition, the role of GBV in the HIV testing to care continuum is less clear. Clarifying how GBV may act as a barrier to accessing HIV services, treatment and care - such as anti-retroviral treatment (ART) or pre-exposure prophylaxis (PrEP) - will not only provide insights into how to best meet individual women’s HIV care needs, but also inform public health oriented HIV epidemic control strategies.

**Methods:**

Through a comprehensive scoping review, we synthesized and analyzed existing evidence regarding the influence of GBV on engagement in PrEP and the HIV care continuum among women living with HIV, including members of key populations (female sex workers, transgender women and women who use drugs). We explored PubMed, Scopus and Web of Science for peer-reviewed studies published in 2003–2017. Of the 279 sources identified, a subset of 51 sources met the criteria and were included in the scoping review.

**Results:**

Studies were identified from 17 countries. The majority of studies utilized quantitative cross-sectional designs (*n* = 33), with the rest using longitudinal (*n* = 4), qualitative (*n* = 10) or mixed methods (n = 4) designs. Taken together, findings suggest that GBV impedes women’s uptake of HIV testing, care, and treatment, yet this can vary across different geographic and epidemic settings. Substantial gaps in the literature do still exist, including studies on the impact of GBV on engagement in PrEP, and research among key populations.

**Conclusions:**

This scoping review contributes to our knowledge regarding the role GBV plays in women’s engagement in PrEP and the HIV care continuum. Findings reveal the need for more longitudinal research to provide insights into the causal pathways linking GBV and HIV care and treatment outcomes. Research is also needed to illuminate the impact of GBV on PrEP use and adherence as well as the impact of GBV on engagement along the HIV care continuum among key populations. It is critical that programs and research keep pace with these findings in order to reduce the global burden of GBV and HIV among women.

## Background

Gender-based violence (GBV), defined as violence perpetrated against an individual based on their gender/gender identity [1, 2], is an important global health and human rights concern. GBV includes physical, sexual and psychological/emotional violence and can be perpetrated by a variety of actors, including intimate partners (referred to as intimate partner violence (IPV)), family members, community members, and representatives of the state (e.g. law enforcement officials) [1–3]. GBV is a common experience for women globally, with estimates suggesting that 1 in 3 women experience some form of GBV in their lifetime, primarily from an intimate partner [4]. Marginalized populations including female sex workers (FSW), transgender women, and women who use drugs experience even higher rates of GBV, often perpetrated by intimate partners and non-partners such as representatives of the state [5–7].

GBV is associated with several acute and long-lasting health consequences [8], including HIV [9, 10]. Globally, women are disproportionately affected by HIV—particularly in the epidemic’s epicenter in sub-Saharan Africa-- and HIV is the leading cause of death among women of reproductive age [11]. FSW, transgender women, and women who inject drugs are at even greater risk for HIV acquisition and HIV-related morbidity and mortality, due to their marginalized status in society, and the associated barriers they face in accessing HIV services [11]. Because of this, members of these populations have been identified as ‘key populations’ by international HIV organizations including the Joint United Nations Programme on HIV/AIDS (UNAIDS) [12].

A large body of evidence has demonstrated an inextricable link between GBV and HIV among women [9, 10]. Women who experience GBV are more likely to engage in HIV risk behaviors such as condomless sex and are more likely to be living with HIV [9, 10, 13]. Similarly, evidence suggests that women living with HIV (WLHIV) are at increased risk for experiencing violence [14–16]. However, the role of GBV in the HIV care continuum (which includes HIV testing, and appropriate care and treatment for HIV or to prevent transmission via PrEP) is less clear. It is critical to clarify how GBV may act as a barrier to accessing HIV testing, linking to and staying engaged in HIV care and treatment, as well as PrEP, not only to address violence against individual women and meet their HIV care needs, but to also achieve public health-oriented HIV epidemic control goals. In 2015, for example, the World Health Organization (WHO) published guidelines which promoted the use of anti-retroviral treatment (ART) by anyone diagnosed with HIV, given the protective effects of early treatment initiation [17–19]. Further, in 2017, UNAIDS adopted the ‘epidemic control’ paradigm whereby the global HIV response is now working towards 90% awareness of HIV status, 90% of those with HIV on treatment, and 90% of those on treatment virally suppressed [20]. Also in 2017, WHO finalized guidelines promoting pre-exposure prophylaxis (PrEP) - a formulation of antiretrovirals (ARVs) that prevents HIV acquisition even if exposed to HIV [21, 22] - for all those at substantial risk of HIV, including members of key populations [18]. In response, a massive global effort to encourage HIV testing and treatment has been rolled out, as testing is the entry-point to HIV care and ART for those living with HIV, as well as PrEP for those at substantial risk of HIV. Key to the success of both HIV treatment and PrEP use is the regular adherence to the medications [23].

Over the past several years, a limited body of evidence has documented GBV as a barrier to women’s engagement in the HIV care continuum. For example, a 2015 systematic review and meta-analysis by Hatcher and colleagues [24] – conducted before the guidelines mentioned above were established - explored the effects of IPV on ART use, ART adherence (measured via self-report and viral load), and retention in HIV care among WLHIV. The review identified 13 cross-sectional studies, primarily from the United States, and the meta-analysis demonstrated that IPV was associated with lower ART use, lower self-reported ART adherence, and lower odds of viral suppression [24]. This review did not include the literature exploring the effect of GBV on HIV testing or PrEP, and none of the identified studies included key populations. The authors noted the need for future research to explore the effect of GBV on the care continuum for these populations [24].

In light of the new global test and start guidelines [18], research is needed to summarize the evidence regarding the role GBV plays in engagement in the HIV testing to care continuum and PrEP among women, including members of key populations. Such a review can provide important insights into areas for future research and possible avenues for intervention. Accordingly, building upon the findings of the 2015 systematic review, we conducted an updated review of the evidence linking GBV to engagement in the HIV care continuum and PrEP using a ‘scoping’ methodology. A scoping review provides a more comprehensive review of the literature than a systematic review by looking broadly across study designs (for more detail, see the methods section) [25]. The present study aimed to expand upon the prior systematic review in two ways. First, we examined the evidence regarding the relationship between GBV and HIV testing as well as PrEP use and adherence, in addition to care and treatment. Second, we sought to identify studies that assessed the effect of GBV on engagement in the HIV care continuum and PrEP among members of key populations, including FSW, transgender women, and women who use drugs.

## Methods

### Scoping review

We conducted a scoping review, which enables researchers to summarize what is known about a certain topic for dissemination to policy makers and practitioners, and to identify gaps in the existing literature [25]. In contrast to systematic reviews, which are guided by a research question focused on a particular study design (typically restricted to quantitative methods), scoping reviews aim to “identify all relevant literature regardless of study design” [25]^(p.22)^. Additionally, scoping reviews call for an iterative process of refining search terms as the researcher becomes more familiar with the literature, to ensure the review is comprehensive [25].

### Identifying the research questions and relevant literature

This scoping review was guided by Arksey and O’Malley’s (2005) methodological framework [25], and examined the known relationship between GBV and engagement in the HIV care continuum and PrEP among women, including members of key populations (FSW, female drug users, and transgender women). When examining the care continuum, we included HIV testing, linkage to and engagement in care, ART adherence and viral suppression. The team identified a search strategy based on a review of the literature and medical subject heading (MeSH) terms. We explored the three search engines (PubMed, Scopus and Web of Science) for studies published in peer-reviewed journals in English between January 2003 and November 2017. We began our search in 2003 given that the WHO and UNAIDS began their initiative to roll out ART in low and middle income countries during that year [26]. Table 1 outlines the search terms used for each search engine. For each database, we conducted separate searches for each population given that individual searches in some cases yield different (and more) articles than a combined search.

**Table 1:** Search terms

Search engine	Search terms
PubMed	((gender based violence [TIAB]) OR (intimate partner violence [TIAB]) OR (violence against women [TIAB]) OR (domestic violence [TIAB])) *AND* ((HIV services [TIAB]) OR (HIV care cascade [TIAB]) OR (HIV treatment cascade [TIAB]) OR (HIV care continuum [TIAB]) OR (Pre-exposure Prophylaxis [TIAB]) OR (HIV test* [TIAB]) OR (linkage to HIV care* [TIAB]) OR (engagement in HIV care* [TIAB]) OR (antiretroviral adherence [TIAB]) OR (viral load [TIAB]))
Scopus	TITLE-ABS-KEY (“gender based violence” OR “intimate partner violence” OR “violence against women” OR “domestic violence”) *AND* TITLE-ABS-KEY (“HIV services” OR“HIV care cascade” OR “HIV treatment cascade” OR “HIV care continuum” OR “Pre-exposure prophylaxis” OR “HIV test*” OR “linkage to HIV care*” OR “engagement in HIV care*” OR “antiretroviral adherence” OR “viral load”)
Web of Science	TS = (“gender based violence” OR “intimate partner violence” OR “violence against women” OR “domestic violence”) *AND* TS = (“HIV services” OR “HIV care cascade” OR “HIV treatment cascade” OR “HIV care continuum” OR “Pre-exposure prophylaxis” OR “HIV test*” OR “linkage to HIV care*” OR “engagement in HIV care*” OR “antiretroviral adherence” OR “viral load”)

### Selecting the literature

We reviewed the titles and abstracts of all identified sources. The team created *‘*post hoc*’* exclusion criteria at this point to further narrow the review. Developing post-hoc exclusion criteria is a hallmark of the scoping review methodology. It is recommended to maximize the likelihood that researchers identify all relevant criteria as they familiarize themselves with the literature [25]. We excluded articles that were opinion pieces, protocols describing study designs, and literature reviews (although we did include individual studies that were referred to in literature reviews that met our inclusion criteria). We also excluded papers that explored violence and only the acceptability or awareness of HIV services, as our focus was on the influence over behaviors. Finally, if papers included data from both male and female participants, we excluded those that did not disaggregate the results by sex. The articles of the remaining sources were reviewed in full. Reference sections of all included sources were hand searched for additional relevant sources not already identified by the database search. Relevant sources were included in the full review.

### Charting, collating and summarizing the information

The first author created a matrix to chart relevant information about all the sources reviewed. Specifically, the chart included details about the study design, sample size, population and relevant findings. In accordance with Arksey and O’Malley’s framework [25], the research team held meetings to discuss the overall themes emerging from the reviewed literature and to identify gaps in the literature that warranted further exploration.

## Results

**Fig. 1:**
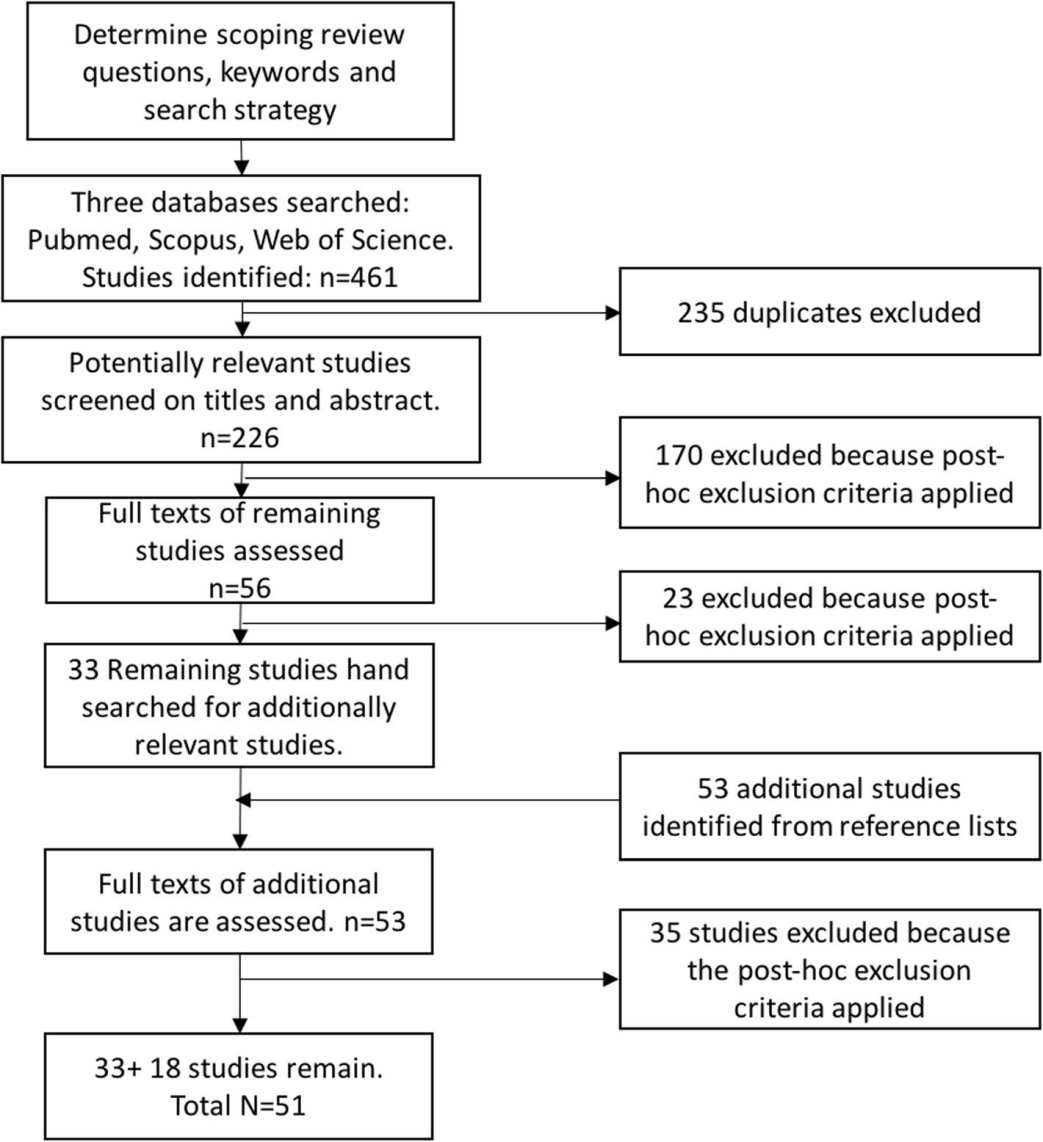
Flow diagram of review process

As shown in Fig. 1, the team identified 226 non-duplicate sources to review from the database search. An additional 53 sources were added after reviewing the reference sections of the sources identified by the database search. Of these 279 sources, a subset of 51 sources were included in the scoping review (Table 2). Studies were from 17 countries: 10 countries from Africa (Uganda, Kenya, Zambia, Malawi, Ethiopia, South Africa, Tanzania, Cameroon, Tunisia and Cote D’Ivoire), three countries from Asia (Nepal, India, Malaysia), two countries from South America (Dominican Republic and Bolivia), and North America (United States (U.S.) and Canada). The majority of studies identified utilized quantitative cross-sectional designs (*n* = 33), four used quantitative longitudinal designs, ten were qualitative and four were mixed methods. Below, we outline the evidence regarding the effects of experiences of violence on women’s engagement in the HIV care continuum and PrEP.

**Table 2:** Summary of published literature on GBV as a barrier to women’s uptake of HIV prevention and treatment services and behaviors

Author	Country	Population	Sample size (women)	Design	Findings
HIV TESTING					
Quantitative					
Turan et al. (2011) [27]	Kenya	Pregnant women (≥18 years) attending ANC clinics	1525	Cross-sectional	Anticipated stigma from one’s partner upon testing positive for HIV was significantly associated with increased odds of refusing to test for HIV. The measure of anticipated stigma from one’s partner was based on the combined score from two items; anticipating break-up of marriage or relationship and physical violence from a partner.
Nelson et al. (2016) [28]	Zambia	Women (15–49 years)	5014	Cross-sectional	In the unadjusted analysis, IPV was significantly associated with increased odds of consenting to HIV testing. A stratified analysis showed that there was a significant association between IPV and consent to test for HIV in rural areas but not in urban areas. However, in the multivariable analysis, there was no significant relationship between IPV and consent to test for HIV.
Satyanarayana (2009) [29]	India	Women (18–50 years)	100	Cross-sectional	This study found no significant differences between women who consented to test for HIV and those who did not in terms of their exposure to violence.
Pearlman (2005) [30]	U.S.	Pregnant and post-partum women (≤ 3 months after delivery) enrolled in in a federally funded nutrition program for women, infants and children (WIC)	433	Cross-sectional	Experiencing IPV in the past 12 months was associated with reduced probability of receiving a prenatal HIV test.
Tucker (2003) [31]	U.S.	Women (18–55 years) sheltered and low-income housed	898	Cross-sectional	Women who had ever experienced sexual violence since they were 18 were significantly more likely to have ever tested for HIV.
Nikolova et al. (2015) [32]	Kenya	Heterosexual couples (men and women *N* = 2862)	1431	Cross-sectional	Experiences of sexual violence were not significantly associated with ever having tested for HIV among women.
Mohammed et al. (2017) [33]	Ethiopia	Heterosexual couples (men and women *N* = 420)	210	Cross-sectional	Women who reported ever experiencing physical violence from their partner were significantly less likely to have tested for HIV.
Etudo et al. (2016) [34]	U.S.	HIV-negative adult women (≥18 years)	79	Cross-sectional	Women who experienced emotional, physical, and/or sexual violence were less likely to test for HIV in the past year, report a longer time since their last HIV test, and reported more barriers to HIV testing, compared to women who had not experienced violence.
Rountree et al. (2016) [35]	U.S.	Adult women (≥18 years)	18,917	Cross-sectional	Women who experienced IPV in their lifetime reported higher rates of HIV testing compared to women who had never experienced IPV.
Nasrullah et al. (2013) [36]	U.S.	Non-pregnant adult women (≥18 years)	29,209	Cross-sectional	Women who experienced threatened violence, attempted violence, or unwanted/forced sex were significantly more likely to have ever been tested for HIV compared to women with no IPV history. However, nearly half of the women who experienced IPV had never tested for HIV.
Brown et al. (2013) [37]	U.S.	Adult women (≥18 years)	30,182	Cross-sectional	Survivors of IPV were twice as likely to have had a HIV test, compared to women who had not experienced IPV.
Decker et al. (2005) [38]	U.S.	Female students in 9th and 12th grade	1641	Cross-sectional	Girls who experienced both physical and sexual dating violence were three times more likely to have been tested for HIV or an STI, compared to girls who had never experienced violence.
Loeliger et al. (2016) [39]	Malaysia	Adult women who use drugs (≥18 years)	103	Cross-sectional	Experiences of adulthood IPV was associated with not testing for HIV.
McCall-Hosenfeld (2013) [40]	U.S.	Women (18–45 years)	1420	Longitudinal	IPV in the past 12 months was significantly associated with increased odds of receiving a test for sexually transmitted infections including HIV in the past two years.
Conroy (2015) [41]	Malawi	Heterosexual couples (men and women *N* = 932)	466	Longitudinal	Physical and sexual violence was not significantly associated with HIV testing among women.
Kiarie et al. (2006) [42]	Kenya	Pregnant adult women (≥18 years) who had not yet tested	1638	Longitudinal	Previous domestic violence was not associated with a reduced uptake of HIV-1 counseling and testing, or PMTCT.
Qualitative					
Naju et al. (2012) [43]	Tanzania	Adult married men and women (≥18 years), PLHIV, healthcare providers, HTC counselors and community leaders (*N* = 91)	48	Qualitative IDIs and Focus group discussions (FGDs)	Women described barriers to couples testing include fears of being beaten if a partner tests positive.
Mixed methods					
Washio (2017) [44]	U.S.	Young women (18–29 years) enrolled in WIC	80	Quantitative (cross-sectional) and Qualitative FGDs	In the quantitative analysis, any lifetime experience of IPV was not significantly associated with testing for HIV in the past 6 months. Focus group participants said that fear of a negative reaction from an abusive partner when asking them to get tested or talking about their HIV status was a barrier to accessing HIV testing. They also discussed how mental or emotional abuse may make it difficult for women to go to get tested for HIV.
LINKAGE TO & ENGAGEMENT IN CARE AND TREATMENT					
Quantitative					
Blank et al. (2015) [45]	U.S.	Women of color living with HIV	587	Cross-sectional	Experiences of IPV were not significantly associated with retention in HIV care or viral suppression.
Blackstock (2015) [46]	U.S.	Women of color living with HIV	748	Cross-sectional	Experiences of IPV were not significantly associated with engagement in HIV care.
Dale et al. (2014) [47]	U.S.	WLHIV	138	Cross-sectional	There was no significant main effect of current abuse, history of abuse, or multiple abuses on ART adherence, CD4+ cell count, or HIV viral load. However, among WLHIV who reported experiences of sexual abuse or multiple abuses, resilience was associated with increased odds of ART adherence.
Hatcher et al. (2012) [48]	Kenya	WLHIV (≥18 years)	483	Cross-sectional	Women who anticipated a violent response from their partner were less likely to link to care.
Hampanda et al. (2016) [49]	Zambia	Pregnant and post-partum WLHIV (≥18 years)	320	Cross-sectional	IPV was associated with decreased odds of PMTCT adherence during and after pregnancy.
Sullivan et al. (2015) [50]	U.S.	Women of color living with HIV	563	Cross-sectional	Explored the effect of substance abuse, violence and HIV/AIDS (SAVA syndemic) on viral load. SAVA scores included measures of substance abuse, binge drinking, IPV, poor mental health and sexual risk taking. The study demonstrated that higher SAVA scores were significantly associated with reduced odds of viral suppression.
Trimble et al. (2013) [51]	U.S.	WLHIV receiving care at a HIV clinic	272	Cross-sectional	IPV was associated with reduced ART adherence. IPV was also associated with more detectable viral loads.
Siemieniuk et al. (2013) [52]	Canada	WLHIV (≥18 years) receiving care at a HIV clinic	339	Cross-sectional	IPV was associated with decreased use of ART, and increased interruptions in HIV care longer than one year.
Schafer et al. (2012) [53]	U.S.	PLHIV (women and men) (≥18 years) receiving care at a HIV clinic (Total *N* = 251)	64	Cross-sectional	Found no significant relationship between experiences of IPV and no show rates to HIV clinic appointments among women.
Illangasekare et al. (2012) [54]	U.S.	WLHIV (≥18 years)	196	Cross-sectional	Experiences of IPV were not significantly associated with current ART use, CD4 cell count or HIV-1 RNA levels.
Rose et al. (2010) [55]	U.S.	African American WLHIV (≥18 years) receiving care at a HIV clinic	40	Cross-sectional	Women who experienced IPV had lower CD4 counts and increased HIV viral load. Medication adherence mediated the relationship between IPV and low CD4 count and high viral load.
Lopez et al. (2010) [56]	U.S.	Adult HIV seroconcordant and serodiscordant couples	190	Cross-sectional	ART adherence was negatively associated with experiences of violence among women.
Espino et al. (2015) [57]	U.S.	African American WLHIV (≥18 years)	102	Cross-sectional	Women with a history of violence were less likely to be virally suppressed.
Kidman et al. (2018) [58]	South Africa	Perinatally infected female youth living with HIV (13–24 years)	129	Cross-sectional	Experiences of violence in the past year was associated with poor ART adherence. However, neither lifetime or past year IPV was associated with viral load.
Kacanek et al. (2016) [59]	U.S.	Perinatally infected male and female youth living with HIV (8–15 years) (Total *N* = 268)	142	Cross-sectional	Among girls, indirect violence exposure (i.e. witnessing violence) was not significantly associated with unsuppressed viral load.
Cohen et al. (2004) [60]	U.S.	WLHIV (≥18 years)	1219	Longitudinal	Women who experienced any physical or sexual abuse were significantly more likely to be non-ART users after three month follow up.
Mendoza et al. (2017) [61]	Dominican Republic	Female sex workers living with HIV	268	Cross-sectional	Violence from an intimate partner in the past six months was associated with not currently being on ART and missing an ART dose in the last four days. Violence from a client in the past six months was associated with never having received HIV care and ever-interrupting ART.
Lyons et al. (2017) [62]	Cote D’Ivoire	Adult female sex workers living with HIV (≥18 years)	466	Cross-sectional	Physical and sexual violence were not significantly associated with HIV testing, or ART adherence.
Machtinger et al. (2012) [63]	U.S.	Adult women and transgender women living with HIV (≥18 years)	113	Cross-sectional	Participants who reported recent trauma had over four-times the odds of ART failure, compared to those without recent trauma.
Kalokhe et al. (2012) [64]	U.S.	Inpatient adult male and female crack cocaine users living with HIV (*N* = 343)	173	Cross-sectional	IPV was associated with significantly lower current ART use among females.
Wechsberg et al. (2017) [65]	South Africa	Adult substance using black African women	641	Cross-sectional	Women who reported experiencing physical violence in the past year were significantly more likely to be newly diagnosed with HIV. Experiences of physical or sexual assault in the past year were not significantly associated with ART use.
Qualitative					
Mepham et al. (2011) [66]	South Africa	Pregnant WLHIV (≥18 years)	43	Qualitative IDIs	Women revealed that threats of violence and actual experiences of IPV were a barrier to PMTCT adherence.
Hatcher et al. (2014) [14]	South Africa	Pregnant WLHIV (≥18 years), pregnant abused women, healthcare providers, district health managers (Total *N* = 38)	18	Qualitative IDIs and FGDs	Experiences with IPV limited women’s ability to adhere to PMTCT because taking the medication or accessing HIV services might unintentionally alert male partners to women’s HIV status.
Zunner (2015) [67]	Kenya	WLHIV, Health care providers, Community health workers and community advisory board (Total *N* = 61)	25	Qualitative IDIs and FGDs	Emotional distress from experiences of violence was described as a cause of HIV treatment default through various avenues including loss of appetite and weight loss, which interferes with ART adherence, as well as feelings of hopelessness, which participants said caused women to intentionally stop ART. Women also stated that the emotional distress from violence caused their health to deteriorate, including decreasing CD4 counts, even if they were fully adherent to ARTs.
Hatcher et al. (2016) [68]	South Africa	Pregnant and postpartum WLHIV (≥18 years)	32	Qualitative IDIs	Experiences with IPV led some women to feel depressed, which caused them to unintentionally or intentionally miss PMTCT doses. Women who intentionally missed medication used it as a form of “passive suicidality” to escape IPV.
Conroy et al. (2017) [69]	South Africa	Heterosexual couples with at least one partner living with HIV (Total *N* = 24)	12	Qualitative IDIs	Findings illuminate how relationship conflict, including violence, can result in forgetfulness to take ART pills among women.
Lichtenstein et al. (2006) [70]	U.S.	WLHIV (≥18 years) attending a public health clinic	64	Qualitative IDIs and FGDs	Women who experience violence were unwilling to keep their appointments if they were afraid of their partners, feeling depressed, feeling “too worn down,” or if they were embarrassed by their abuse. Some women reported that their partners prevented them from seeking care, keep appointments, or take their ARTs.
Watt et al. (2017) [71]	South Africa	WLHIV with a history of sexual assault	15	Qualitative IDIs	Women reported how the sexual assault they experienced led to a delay in initially linking to HIV care. Women described how they were emotionally unable to accept their HIV diagnosis after experiencing sexual assault, causing them to delay linking to HIV care. Women also reported how experiences of sexual assault limited their engagement in HIV care and treatment. Specifically, participants described how taking antiretroviral therapy sometimes brought up memories of their sexual trauma history, especially if they acquired HIV through sexual assault.
Kosia et al. (2016) [72]	Tanzania	WLHIV with a history of GBV	35	Qualitative IDIs	Participants described how their male partners verbally abused them, prevented them from attending their HIV care appointments, and threw away their antiretroviral medication. Women reported that such actions prevented them from staying engaged in HIV care and adhering to their antiretroviral therapy.
Maeri et al. (2016) [73]	Kenya & Uganda	Community members, care providers and community leaders from 8 communities (Total *N* = 194)	112	Qualitative IDIs	WLHIV anticipated violent reactions from their partners upon disclosure of their sero-status. As a result, these women avoided disclosing their status to their partners. Non-disclosure was reported as a major barrier in the uptake of HIV care and treatment. For example, some women forgot to take their antiretroviral medication because they hid it outside the home so their husbands would not suspect them of being HIV-positive. Some women reported experiencing physical abuse from their partners upon disclosing their HIV-positive status. Violent reactions typically occurred in the context of sero-discordance.
Mixed Methods					
Orza et al. (2017) [74]	Bolivia, Cameroon, Nepal, Tunisa	WLHIV	197	Quantitative (cross-sectional) and qualitative open-ended survey responses	Fear and experiences of violence prevented women from disclosing their HIV status, which participants said led to anxiety, missing HIV care services, and lower adherence. Women also described facing discrimination and violation of their rights to health from health care providers both in the context of HIV care and treatment and labor and delivery.
Wilson et al. (2016) [75]	Kenya	Female sex workers (FSW) living with HIV (≥18 years)	195	Quantitative (longitudinal) and qualitative IDIs and FGDs	Longitudinal quantitative data analysis revealed that IPV was associated with significantly lower risk of detectable viral load. In the qualitative findings, women did not suggest that experiences with IPV limited their ability to engage in HIV care, initiate and adhere to ART. Women employed different strategies to ensure experiences with violence did not interfere with their engagement in care. Such strategies included not disclosing their HIV status to their partner, and seeking support from their friends or HIV support groups after an episode of IPV to ensure they continued taking their medication.
PRE-EXPOSURE PROPHYLAXIS (PrEP)					
Mixed methods					
Roberts et al. (2016) [76]	Uganda	HIV-negative adult women (≥18 years) in sero-discordant relationships	1785	Quantitative (longitudinal) and Qualitative In-depth interviews (IDIs)	In the longitudinal quantitative analysis, women who reported experiencing IPV in the past three months had increased risk of low adherence to PrEP, measured by pill count and plasma tenofovir. Verbal, economic and physical IPV were associated with low adherence to PrEP. In the qualitative interviews, women reported that IPV caused them to forget their pills, that their partners threw away their pills, and that they would leave their pills behind if they fled the house during a violent episode.

### GBV and its implications for engagement in the HIV care continuum and PrEP

#### HIV testing

The review yielded 19 quantitative studies (three longitudinal and 16 cross-sectional studies), one qualitative study and one mixed method study that explored the relationship between violence and HIV testing among women. Three studies were conducted among members of key populations (two studies were among women who use drugs and one was among FSW). We did not find any studies that examined the relationship between GBV and HIV testing among transgender women.

Results were mixed. A number of the studies found that experiences of violence were associated with reduced HIV testing among women [30, 33, 34, 36, 39]. Qualitative studies described how fear of a violent reaction from one’s partner in the event of a positive test result contributed to reduced rates of HIV testing [43, 44]. A cross-sectional study by Turan et al. (2011) supported these findings by demonstrating that anticipated stigma (defined as break-up of marriage/relationship and physical violence from a partner) upon testing positive for HIV was associated with refusing to test for HIV [27].

Two studies among key populations also found a negative relationship between GBV and HIV testing [39, 65]. A cross-sectional study among women who use drugs in Malyasia found that experiences of adulthood violence from a partner were associated with failure to test for HIV [39]. Another study among substance-using black South African women found that those who experienced physical violence were less likely to be aware of their HIV-positive status [65].

At the same time, five additional studies, all from the U.S., found experiences of violence to be associated with increased HIV testing [31, 35, 37, 38, 40]. One of these studies was a longitudinal study, which found that experiences of intimate partner violence (IPV) in the past 12 months at baseline was significantly associated with increased odds of receiving a test for sexually transmitted infections (STIs) (including HIV) during a two-year follow up period [40].

Seven studies found no significant relationship between experiences of violence and uptake of HIV testing among women [28, 29, 32, 41, 42, 44, 62]. Two of these studies utilized longitudinal designs. Conroy et al. (2015) found that physical and sexual violence at baseline was not significantly associated with receiving a subsequent HIV test during a 16-month follow up period among women in heterosexual couples in Malawi [41]. A cross-sectional study among FSW in Côte d’Ivoire also found no significant relationship between physical or sexual violence and uptake of HIV testing [62].

#### Linkage to and engagement in HIV care

The review yielded thirteen studies (six quantitative, five qualitative, and two mixed-methods studies) that explored the relationship between GBV and linkage to and engagement in HIV care among WLHIV. Two of these studies were conducted among FSW. We did not find any studies that examined this relationship among women who use drugs, or transgender women.

The research suggests that experiences of violence are associated with reduced linkage to HIV care among WLHIV [48, 71, 73], including FSW [61]. A cross-sectional study among WLHIV in Kenya found that women were less likely to link to HIV care if they anticipated a violent reaction from their partner upon learning the woman’s HIV-positive serostatus [48]. This was supported by qualitative research from Uganda and Kenya, which found that women avoided disclosing their HIV-positive status to their partner because they feared a violent reaction [73]. Women revealed that non-disclosure was a major barrier to uptake of HIV care because they did not want to inadvertently disclose their status to their partner by seeking care [73].

There is also some evidence to suggest that GBV prevents WLHIV from staying engaged in HIV care, once they have already enrolled [52, 70–72]. A cross-sectional study among WLHIV in Canada found that experiences of IPV were associated with increased interruptions in HIV care longer than one year [52]. Qualitative evidence suggests that women skip their HIV care appointments due to fear that attending such appointments will unintentionally alert their partner to their HIV-positive status and result in violence [70]. Having partners who threaten women with violence or prevent them from attending their HIV care appointments may also prevent WLHIV from staying engaged in HIV care [70, 72]. Furthermore, women who experience violence may miss their appointments due to depression, physical illness, or injury caused by violence, and shame of being abused [70, 71].

Three other studies found no significant relationship between experiences of violence and engagement in HIV care among WLHIV [45, 46, 53]. All three studies were cross-sectional and from the U.S. Additionally, a mixed methods study among FSW in Kenya found that GBV did not limit women’s engagement in HIV care [75]. Findings from this study suggest that women utilized a number of different strategies to stay engaged in HIV care including not disclosing their HIV status to their partner and seeking support from their friends.

#### Antiretroviral therapy initiation and adherence

We identified 29 studies that explored the relationship between GBV and ART initiation and adherence among WLHIV. Eighteen studies were quantitative (17 cross-sectional and one longitudinal), 9 were qualitative, and two utilized mixed methods. Six of these studies were conducted among members of key populations: three among FSW [61, 62, 75], two among women who use drugs [50, 64], and one among transgender women [63].

Taken together, evidence suggests that WLHIV who experience violence are significantly less likely to initiate [60] and adhere to ART [14, 49, 51, 52, 56, 58, 61, 64, 66, 68, 69, 72, 73], and ultimately achieve viral suppression [50, 51, 55, 57, 63]. In terms of ART initiation, a longitudinal study among WLHIV in the U.S. found that women who experienced physical or sexual violence were significantly more likely to be non-ART users after a three-month follow-up period [60]. In a cross-sectional study, Espino et al. (2015) found that African American women in the U.S. with a history of violence were significantly less likely to be virally suppressed than women without a history of violence [57]. Hampanda et al. (2016) found that violence from a partner was associated with reduced adherence to PMTCT during and after pregnancy among pregnant and post-partum WLHIV in Zambia, also assessed cross-sectionally [49].

When looking specifically at key populations, a cross-sectional study among FSW living with HIV in the Dominican Republic found that experiencing violence from a non-paying intimate partner was associated with not currently being on ART and missing a recent ART dose [61]. Kalokhe et al. (2012) found that experiences of IPV was associated with significantly lower current ART use among female crack cocaine users in the U.S. [64]. Another study found that women of color from the U.S. with higher levels of substance abuse, binge drinking, IPV, poor mental health, and sexual risk taking had reduced odds of viral suppression [50]. Finally, Machtinger et al. (2012) found that recent trauma (defined as having been abused, threatened, or the victim of violence in the past 30 days) was associated with having a detectable viral load among both cis-gendered and transgender women [63] .

Qualitative studies shed light on potential mechanisms through which GBV can lead to poor ART adherence. Evidence suggests that women may choose to keep their HIV-positive status a secret from their partner because they fear their partner may become violent upon learning their HIV status [14, 73, 66, 68, 74]. As a consequence, women hide their pills and have to take their medication in secret [14, 68, 73, 74]. This sometimes leads to missed doses of ART [14, 73, 74]. Additional qualitative research has revealed that some women’s partners throw away their ART medication, or otherwise prevent them from taking their ART medication, which limits their adherence [70, 72]. Other research has demonstrated that WLHIV who experience violence from their partners can skip treatment due to depression or feelings of hopelessness [67–69].

Although the majority of evidence in the literature suggests a negative association between violence and ART use and adherence, we did find six studies among WLHIV that did not follow this trend [45, 46, 54, 59, 62, 75]. For example, a cross-sectional study of WLHIV attending an HIV clinic in Baltimore, Maryland did not find a significant association between GBV and current ART use, CD4 cell count, or HIV-1 RNA levels (which were used as a proxy for ART adherence) [54]. The authors argue that this finding may be due the fact that women in the sample were recruited from an HIV clinic and, therefore, all participants were engaged in HIV care and treatment [54]. As another example, a mixed methods study among FSW living with HIV in Kenya found that GBV was associated with significantly lower risk of a detectable viral load [75]. Women participating in the qualitative component described how they used different strategies, such as keeping their status secret from their abusive partner, to ensure that they were not prevented from accessing care [75]. Additionally, a cross-sectional study among FSW in Cote D’Ivoire did not find a statistically significant relationship between experiences of violence (physical or sexual) and ART adherence [62].

#### Pre-exposure prophylaxis (PrEP)

We found only one study that examined the relationship between GBV and PrEP use and adherence. Roberts et al. (2016) conducted a mixed-methods study among serodiscordant couples in Uganda, which found that HIV-negative women who experienced IPV in the past three months had a significantly increased risk of low adherence to PrEP by pill count and by plasma tenofovir, compared to women who had not experienced violence [76]. In qualitative interviews, HIV-negative women described how conflict in their homes made it difficult for them to remember to take their PrEP pills [76]. Others reported escaping their homes after a violent episode and forgetting to take their PrEP pills with them, and, as a result, they missed some doses [76].

We were unable to find studies that examined the relationship between experiences of violence and PrEP use among FSW, transgender women, or women who use drugs.

## Discussion

Taken together, findings from this wide-ranging examination of recent literature suggest that GBV impedes women’s engagement in biomedical HIV prevention, care, and treatment services. We see a similar relationship for female members of key populations, with a small but growing evidence base. Several studies suggest that women who experience violence are less likely to link to HIV care [48, 61, 71, 73], initiate and adhere to ART [14, 49, 51, 52, 56, 58, 60, 61, 64, 66, 68, 69] and less likely to achieve viral suppression [50, 51, 55, 57, 63]. Qualitative evidence suggests women avoid disclosing their HIV-positive status to their partner because they fear their partner may become violent upon learning their HIV status [14, 66, 68, 73, 74]. Such non-disclosure, was highlighted as a barrier to engagement in HIV care and treatment, due to fear of inadvertently alerting one’s partner to one’s HIV positive status, and potentially experiencing violence [14, 68, 73, 74]. Other research suggests a mental health pathway linking experiences of GBV to sub-optimal engagement in HIV care and treatment [67–69].

However, evidence specifically regarding the relationship between GBV and uptake of HIV testing varied across settings. Several studies from high-income countries, for example, found that GBV was associated with increased testing [31, 35–38, 40], while most studies from low and middle-income countries revealed no significant relationship between GBV and HIV testing [28, 29, 32, 41, 42]. While these studies were for the most part cross-sectional, and therefore do not indicate whether experiences of GBV led to increased testing or vice versa, it is feasible that the positive relationship between experiences of GBV and HIV testing found in high-income countries may in fact reflect increased perceived risk of HIV among survivors [77] and an interest in ascertaining HIV status, as well as enhanced infrastructure in these settings to address and respond to GBV.

There were several notable remaining gaps in the literature. We only found one study that examined the relationship between experiences of GBV and engagement in PrEP [76]. This study was from Uganda, and there were no such studies from higher-income countries, where PrEP is more accessible and established as an HIV prevention strategy. Additional research is needed, including longitudinal studies, to further understand the role that GBV plays in engagement in PrEP. The need for such evidence will become increasingly pronounced as PrEP is introduced and scaled up in the Global South.

Information about the effects of GBV on engagement along the HIV care continuum for female key populations living with HIV was also limited, and thus additional research is sorely needed. Furthermore, we did not find any study that explored the relationship between GBV and PrEP use among female key populations. It is critical to understand how experiences of GBV may influence these populations’ access to PrEP, especially given their heightened risk for both GBV and HIV [6, 7, 11, 78], and the 2017 WHO guidelines highlighting the need for PrEP among these populations [18].

It is also important to note the dearth of longitudinal studies that have explored the relationship between GBV and women’s engagement in biomedical HIV prevention, care and treatment. This review identified only four longitudinal studies. Three of these studies examined the relationship between GBV and HIV testing, and one assessed the effect of GBV on ART use. We did not identify any longitudinal studies that examined the relationship between experiences of GBV and linkage to and engagement in HIV care as well as PrEP use or adherence. Longitudinal studies are urgently needed to better understand the effect of GBV on engagement in biomedical HIV prevention, care and treatment to inform future intervention work.

Given the existing evidence highlighted in this review suggesting that GBV is an important barrier to engagement in the HIV care continuum, programmatic responses are urgently needed. However, our review only identified one paper describing an evaluation of a program that explicitly sought to address GBV with the goal of improving any HIV care continuum outcomes [79]. Collins et al. (2017) used a quasi-experimental design to assess the effects of a muti-component intervention, “Creating Lasting Family Connections,” among African American women in the U.S. [79]. This intervention sought to address factors that place African American women at increased risk for HIV including substance use and violence. Intervention modules promoted positive relationship and parenting skills, conflict resolution skills, offered guidance on how to incorporate substance use and violence prevention messages into activities with children, and improved knowledge about HIV transmission and substance abuse. Participants in the intervention were significantly more likely to test for HIV, and significantly less likely to report IPV in the past 3 months, relative to the comparison group [79]. We did not come across literature describing evaluations that found effects on engagement in HIV care or treatment, or uptake and adherence to PrEP among HIV negative women.

We do acknowledge that a number of studies have evaluated the effects of interventions that have sought to address GBV and HIV risk [80–83]. Researchers could draw upon related lessons learned to address GBV and engagement in HIV care and treatment. For example, several past interventions have aimed to mitigate and transform inequitable gender dynamics (such as support for ‘toxic’ masculinities, and unequal power in relationships) that can lead to GBV and HIV risk behaviors [81, 83, 84]. Future research could explore the impact of promoting gender equitable norms on both GBV and engagement in the HIV care continuum and PrEP. Other researchers have integrated GBV screening into HIV testing services to identify survivors of violence, who might otherwise not report their experience, and offer support and information [85, 86]. It is also possible that HIV care appointments could provide an important opportunity for health care providers or counselors to screen women for GBV and strategize ways to prevent future violence and stay engaged in HIV care and treatment.

This scoping review was limited to articles written in English, and as such we may have excluded relevant articles written in other languages. Additionally, while we recognize that both women and men experience GBV, this review is focused on violence experienced by women, and the HIV service experiences of women. As such, this study does not address what is known about GBV and engagement in the HIV care continuum and PrEP among men, including men who have sex with men. This area certainly warrants further research.

## Conclusion

The linkages between GBV and HIV acquisition have been documented since the early 2000s. This review presents the latest evidence on how GBV can also impede the uptake of HIV testing, care and treatment and PrEP. Findings suggest that the relationship between experiences of GBV and sub-optimal engagement in the HIV care continuum is also significant, although it can vary by geographic context and epidemic setting. However, this review highlighted important gaps in the literature including a dearth of research on the role GBV plays in PrEP use and adherence, limited research on the effect of GBV on engagement in HIV care and treatment and PrEP among members of key populations, and very few longitudinal studies. Future research should prioritize addressing these gaps in the literature.

The global HIV response continues to evolve at an extraordinary pace, with new biomedical strategies creating the potential for epidemic control on the foreseeable horizon. It is critical, however, that programs and research keep pace with these changes by continuing to train a critical lens on gender inequity—and GBV as a particularly severe sequela—as a persistent driver of HIV. Only by continuing to place women at the center of the global HIV response will we achieve the ambitious UNAIDS HIV epidemic control goals.

## Data Availability

Not applicable.

## Abbreviations

ART: Antiretroviral therapy
ARVs: Antiretrovirals
FSW: Female sex workers
GBV: Gender based violence
IPV: Intimate partner violence
PrEP: Pre-exposure prophylaxis
U.S.: United States
UNAIDS: Joint United Nations Program on HIV/AIDS
WHO: World Health Organization
WLHIV: Women living with HIV
